# Are Community Pharmacists Ready to Deliver Pharmacogenomics Testing Services?

**DOI:** 10.3390/pharmacy11060170

**Published:** 2023-10-26

**Authors:** Heba Ghazal, Ayaz Tahar, Thuy Mason

**Affiliations:** School of Life Sciences, Pharmacy and Chemistry, Kingston University London, Kingston Upon Thames, London KT1 2EE, UK

**Keywords:** community pharmacist, pharmacogenomics, pharmacogenomics testing, knowledge, training

## Abstract

This study assesses the readiness and willingness of community pharmacists in England to deliver the pharmacogenomic (PG) testing service. A survey covering demographics and four key themes including awareness and training, general views and experience, barriers, willingness, and confidence was distributed to community pharmacies in the boroughs of Croydon and Sutton in South London. A total of 51 pharmacists responded to the survey. The study revealed that most respondents had a limited familiarity or understanding of pharmacogenomics (*n* = 32, 63%). Moreover, on average, around 60% of participants were unable to accurately identify drugs that currently have or could have potentials for PG testing. They indicated that their pharmacogenomic education and training is inadequate, with only 2/51 pharmacists reported receiving relevant training. Time constraints, shortage of staff and lack of knowledge were identified as barriers that could hinder the implementation of PG. Over 60% of respondents expressed willingness to provide PG testing service after receiving adequate training. The study found that currently not all community pharmacists are prepared to provide PG testing services, with newly qualified pharmacists appearing to have an upper hand when it comes to understanding the subject. Therefore, consistent, and uniform training is required to allow community pharmacists with all years of experience to equally contribute to the implementation of PG testing.

## 1. Introduction

### 1.1. Background

As the human population has evolved, there has been a substantial amount of genetic variation accrued over time. These variations in the genomes have a significant impact on inter-individual differences. Hence, it is ascertainable that no two human beings, even twins, share the same genetic makeup. There are different forms in which genetic variation can happen including deletions, insertions, copy number variations and single nucleotide polymorphisms (SNPs) [1]. There are approximately 3.2 billion base pairs in the human genome which form a sequence of nucleotide bases, these form genes, which then form chromosomes. The majority of genetic sequences are the same between individuals but due to the historical process of mutations and genetic drift, some of the population carry one variant of a gene (allele) while others carry a different allele. When this allele is comprised of a single change to a nucleotide, it is labelled as a Single Nucleotide Polymorphisms (SNP). SNPs are considered to be responsible for variation of traits that are visible e.g., skin or eye colour, as well as traits that may not be so visible e.g., how predisposed someone is to disease or their response to medications [2]. There are an estimated 20,000 protein-coding genes that determine the size, shape and configuration of every protein within living organisms, and humans are estimated to make up to one million proteins. These proteins can act as enzymes, transporters, drug receptors, binding sites, structural components to cells and hormones; they are pivotal to almost every pharmacological and biochemical reaction taking place within the body. Pharmacogenes are those genes that are responsible for coding proteins that are involved in the metabolism, action, and toxicity of drugs. Protein function is very much dependent on conformational shape and a deviation in amino acid sequence, can influence how it is folded, hence affecting biological activity [3].

Pharmacogenomics (PG) targets specific gene variations that patients possess, to facilitate medication selection and predict drug response [4]. The notion that pharmacogenomics has a potential to improve diagnostics, cure diseases at a molecular level and improve the understanding of drug metabolism on a genetic level, has become more realistic since the human genome project in 2003 [5]. One such example that led the way within the UK in 2005 was abacavir [6]. This drug is used in the treatment of Human Immunodeficiency Viruses (HIV) infection and is classified as anti- HIV medication known as a nucleoside reverse transcriptase inhibitor. Approximately, 5–8% of the general population can develop hypersensitivity reactions to this medication usually within the first 6 weeks, which can then lead on to a life-threatening reaction or even results in death. It was then found that patients who have an allele known as HLA-B*57:01 are at the highest risk of developing this hypersensitivity reaction and in such instances abacavir must be avoided [7]. Subsequently, it is compulsory to screen patients for HLA-B*57:01 to minimise the risk of any adverse reactions, which concludes, those patients that test positive for HLA-B*5701 should be given alternative treatment, consequently decreasing the occurrence of fatal outcomes [6].

An example of a consequential SNP is within an allele of the gene *CYP2C19*. This allele, known as CYP2C19*2, is a product of the substitution of an adenine [A] instead of a guanine [G] at nucleotide 681 (i.e., c.681G > A) within the coding segment of the gene and synonymously impacts the amino acid proline at position 227. This mutation results in a non-functional enzyme [8]. Clopidogrel is one of the most prescribed antiplatelets used to reduce the risk of myocardial infarction and stroke. It is a prodrug that requires activation via the enzyme *CYP2C19* and patients who carry one or two copies of the allele CYP2C19*2 are poor metabolisers. This results in a reduced inhibition of platelet activation, which proliferates the risk for major cerebrovascular and cardiovascular occurrences, hence putting the patient at risk and it is advised they are put on an alternative antiplatelet instead [9]. Recently, the National Institute for Health and Care Excellence (NICE) has released new draft guidance suggesting that clinicians should provide genotype testing to individuals prescribed clopidogrel to identify patients who may have a genetic predisposition that affects their response to this drug and this approach is regarded a cost-effective measure [10]. Implementation of pharmacogenomics testing in the National Health Service (NHS) in England has been relatively limited, with an example of testing for patients diagnosed with colorectal and breast cancer, where a test is offered to identify the *DPYD* variant prior to initiating 5-fluorouracil chemotherapy treatment.

### 1.2. Community Pharmacists and Pharmacogenomic Testing

The role of community pharmacists has advanced over the past 10–15 years, from the traditional role of dispensing medicines to the introduction of services led by pharmacists, ranging from, community pharmacist consultation service, vaccination services, healthy living and more. By offering an array of services, community pharmacists play an important role in improving patients’ access to healthcare and optimising their treatment outcomes. In addition, pharmacists provide counselling to patients before they start a new medication, hence making it ideal for them to offer a PG testing service. The contribution of pharmacists in offering these services, as accessible healthcare providers, have shown an acceptability by the public. The testing service in community pharmacy would entail an individual taking a saliva cheek swab, which contains their DNA, and sending it to the laboratory for testing. Once the results are received, community pharmacists can discuss any concerns with the prescriber after reviewing the patient’s medications [11].

In a study conducted in the USA, 101 independent community pharmacists were surveyed and 75% expressed interest in offering a personalised medicines service. However, when they were asked to describe their knowledge of PG and their readiness to implement this service, more than 50% expressed having poor knowledge in the subject. They also mentioned that they would not feel comfortable in making prescribing recommendations to physicians or providing counselling to patients based on the results of the genetic screening without additional education and training [12]. Similarly, a qualitative study conducted in the Greater Pittsburgh area showed that enriched PG education and training, along with active learning to build confidence, reputable clinical recourses, and access to a network of experts within the PG field, were requirements of experienced pharmacists to be able to implement and carry out a successful PG testing service. Thus, understanding pharmacists’ perceptions on their need for education and training can help to boost the clinical implementation of PG services led by community pharmacists [13].

Pharmacists can play a valuable role in genomic testing services, as well as there being a plethora of reasons that underline the need for these services. A Canadian study was carried out to examine the impact of PG testing carried out by PG specialist pharmacists in two busy urban community pharmacies. All eligible patients had their DNA analysed through a buccal swab and genotyping assay followed by devising a therapeutic report based on the patients inherited drug metabolic profile. The pharmacists were able to interpret the results and report any clinically significant therapeutic issues to the physician in charge of the patients care. From the results, it was evident that the primary reasons for PG testing were ineffective therapy, adverse reactions and guiding the initiation of therapy. Overall, among the patients tested, 100% of them had impactful interventions made by the pharmacists, of which 60% of individuals required a change in therapy, 13% needed dosage adjustments, 22% required increased monitoring and in 4% of the cases medication stopping was necessary [14].

Although PG testing has been carried out in hospitals in England for specific medications, there have been delays in its integration into primary care. The launch of PG testing pilot has been recently announced in the community in general practice clinics in the northwest of England in 2023. This pilot programme focusses on a certain group of medicines, namely antidepressants, proton pump inhibitors and statins [15]. This came as an evidence-based notion from the ‘(Preemptive Pharmacogenomic Testing for Preventing Adverse Drug Reactions) PREPARE’ trial, which was carried out in multiple European health-care system settings, including the UK, on a range of different diseases and medicines. The trial revealed and affirmed that genotype-informed treatment can reduce relevant adverse drug reactions by 30% using a 12-gene PG panel to individualise treatment [16]. The benefits of PG testing have been acknowledged by the NHS England in terms of enhancing safety, facilitating quick diagnosis in rare diseases, decreasing the incidence of adverse drug reactions, and enhancing the therapeutic outcomes. The NHS Genomic Medicine Service, in their plans to promote the precision medicine, aims to become a leading national healthcare system in integrating genome sequencing into standard care and completing the sequencing of 500,000 whole genomes by 2023/24 [17]. In accordance with the NHS framework and its needs, recent policy initiatives have underscored the importance of expanding the scope of services offered by community pharmacies and strengthening their integration within the primary care system [18]. These policies stem from the need to utilise pharmacists’ expertise, clinical knowledge, and their accessible services, with the aim of improving patient care and reducing the pressure on other parts of the healthcare system. Consequently, the provision of PG testing through community pharmacists aligns with these objectives.

Hence, analysis of the current literature reveals that community pharmacists can play an integral part in PG testing service. Therefore, the rationale for the current study is to determine the level of awareness and knowledge that community pharmacists have regarding PG and their willingness to embrace the responsibility of delivering this service.

## 2. Materials and Methods

### 2.1. Study Design

A cross-sectional survey was used, comprising 36 questions covering demographics and four key themes including: awareness and training, general views and experience, barriers, willingness, and confidence. To capture a comprehensive overview of the pharmacists’ perspectives through the survey, a variety of questions were incorporated including open-ended and closed-ended questions, multiple choice questions and questions with 5-point Likert-type scales.

### 2.2. Sample Size and Participants

The boroughs of Croydon and Sutton, which are situated in South London, were selected as the study area. To determine the total number of pharmacies in the area, the “find a pharmacy” option on the General Pharmaceutical Council (GPhC) website was used, and the filter for Croydon and Sutton were then applied. This process identified 112 pharmacies in these areas. It should be noted that while there could be more than one regular pharmacist or locum per pharmacy, it was not feasible to ascertain the exact number of pharmacists working at each pharmacy. The sample size was calculated using Raosoft’s calculator [19] with values entered for a 5% margin of error, 95% confidence level and a total population size of 112, which generated a result of 87.

### 2.3. Ethics

This study received an ethical approval from the delegated ethical approval team operating under the ethics committee of the Kingston University, Faculty of Health, Science, Social care and Education. Prospective participants were given an information sheet explaining the research prior to deciding if they wish to complete the survey. Informed consent was obtained from all participants involved in the study and all participant responses were anonymous and confidential.

### 2.4. Pilot and Data Collection

Before the full-scale data collection commenced, a pilot study of the survey was completed to assess how participants would respond to the survey. The pilot involved four practicing pharmacists outside the named boroughs of Croydon or Sutton. The subsequent feedback received from the pilot was that the survey was lengthy, and some of the knowledge assessment questions were difficult. In response to this feedback, the survey underwent a review, during which some questions were removed and an option ‘not sure’ was added to the knowledge section. Pharmacists from various pharmacies within the mentioned boroughs were approached from January to the end of April 2023 and given the Participant Information Sheet. They were asked if they would be willing to participate in the survey. Upon obtaining their consent, they were given the option to complete the survey either using a paper copy or an online form sent to them via email.

### 2.5. Data Analysis

Qualitative and ordinal data (such as Likert scale) were analysed using IBM SPSS Statistics for Windows software (28.0). Descriptive statistics, including percentages, frequencies, averages, and modes were used. Weighted averages were calculated for all Likert-scale questions used within the study. The Fisher Exact Test was used to investigate survey data so that different variables can be compared and a value of *p* < 0.05 was considered statistically significant. For example, the test was used to assess the relationship between duration of pharmacists’ practice (grouped in two groups: less than 10 years and more than 10 years of experience) and their understanding of PG (grouped in two groups: being less familiar and being more familiar with concept of PG).

## 3. Results

### 3.1. Demographic Information

A total of 51 participants responded, giving a response rate of 59%. The majority of respondents 49% (25/51) were between the ages of 23–32 while the minority (4/51) were in the age range of 53–62. The gender distribution showed a slightly majority of females at 57%. The modal length of practice was between 5–10 years. There was an even distribution between those practicing in independent or large pharmacy chains, both at 33% (17/51). Most respondents (*n* = 39) were either regular responsible pharmacists or locums, while only 9 were at managerial level. Table 1 presents the demographic data of the respondents.

### 3.2. Awareness and Training

**Figure 1:**
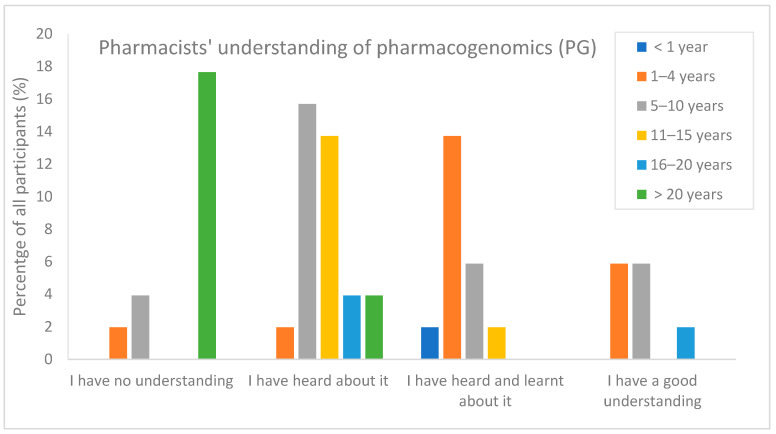
Pharmacists understanding of PG topic in relation to the years of experience (*n* = 51).

Regarding their understanding of pharmacogenomics, 24% (12/51) of respondents indicated they had no understanding of the topic, while only 14% (7/51) claimed to have a good understanding. Among the 11 respondents qualifying over 20 years ago, over 80% (9/11) reported to have no understanding of this topic. On the other hand, among the 13 recently qualified pharmacists (0–4 years), 92% (12/13) acknowledged their previous learning or familiarity with the concept of PG (Figure 1). The association between the number of years of practice and being familiar with the PG topic is considered statistically significant. This is evident from the calculated *p*-value of 0.0004, which is significantly less than 0.05, indicating that pharmacists with more years of experience tend to have a lower level of understanding of the PG topic.

The survey found that only 41% (21/51) of pharmacists were taught about PG during their studies at university. Among those, approximately 57% (12/21) considered it “fairly useful”, while none of the respondents indicated it was highly useful, and 15% (3/21) did not find the contents useful at all. Surprisingly, only 2 out of 51 had received training specific to PG since qualifying as a pharmacist. The type of training received involved E-learning activity and watching training videos. When asked about their preferred method of training, 45% (23/51) chose in-person training while 41% (21/51) preferred online training. Some indicated a preference for shadowing a trained pharmacist (5/51) and only two expressed the preference that university education should be more comprehensive on this topic.

Table 2 displays responses to a question concerning common drugs encountered in practice and whether pharmacists would be able to categorise them as having current PG testing available, or a candidate for future PG testing, or neither. Firstly, when looking at abacavir, over 61% (30/49) of respondents answered correctly by selecting current PG testing available. Only 47% (23/49) of respondents were aware that PG testing for fluorouracil is already being carried out in hospitals prior to initiating this cancer treatment. Similarly, for co-amoxiclav over half correctly classified it as ‘neither’. Conversely, for codeine, carbamazepine and clopidogrel, only 17% (8/49), 33% (16/49) and 33% (16/49) of the pharmacists accurately identified them as suitable for future PG testing, respectively. On average, 44% of respondents selected the answer of “unsure” across the eight medicines assessed.

### 3.3. General Views and Experience

Among the pharmacists who indicated they usually provide recommendations to prescribers regarding drug choice or dose adjustment 69% (35/51), 54% (19/35) stated their suggestions were always or often implemented by the prescriber. However, when specifically inquired about their views of recommendations based on PG testing results, pharmacists had a more positive outlook, with 76% of participants speculated that prescribers would always or often address their recommendations.

**Figure 2:**
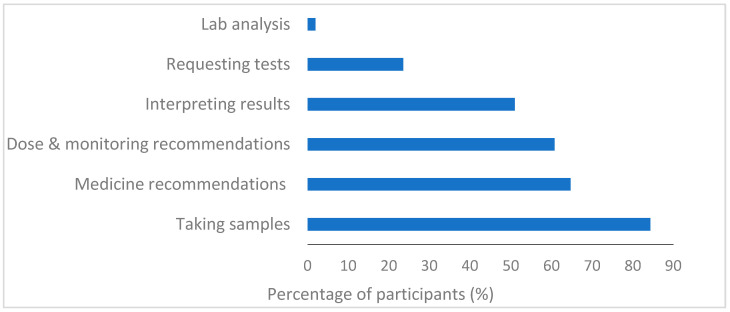
Participants’ perspectives on their role in PG testing (*n* = 51).

In terms of the role of community pharmacists in PG testing, the majority of the respondents 84% (43/51) believe that taking samples would be part of their role. Additionally, more than 60% identified that making recommendations regarding medicines (33/51), dose and monitoring (31/51) would be part of their role. However only half (26/51) agreed that interpreting results would be part of their skillset, indicating a possible lack of self-confidence in their clinical capabilities as community pharmacists (see Figure 2).

### 3.4. Barriers to PG Implementations

**Figure 3:**
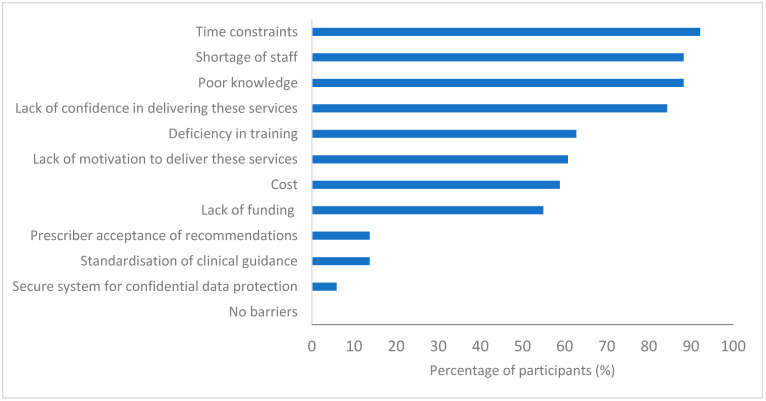
Pharmacists’ views on barriers to implementing PG testing services in community pharmacy (*n* = 51).

With regards to barriers to PG testing implementations, more than 80% of participants agreed that time constraints (47/51), shortage of staff (45/51), poor knowledge (45/51) and lack of confidence (43/51) are the primary obstacles in the implementation of this service. More than half also agreed that a lack of funding (28/51), a deficiency in training (32/51) and a lack of motivation (31/51) were barriers in place. Less than 20% (7/51) of pharmacists believed prescriber acceptance of their recommendations would be a hurdle (Figure 3). Although it is worth noting that currently none of the participants reported having delivered the PG testing service nor having access to standardised guidance for integrating a PG service into practice.

The participants anticipated potential drawbacks if PG testing were to be successfully implemented including an increase in their workload 98% (50/51), patients’ apprehension 35% (18/51), data protection issues 18% (9/51) and disagreement with prescriber 12% (6/51). Furthermore, when asked to rate the likelihood of PG changing their daily practice in the future on a Likert scale (1 = very unlikely and 5 = highly likely), most of the respondents (mean score of 3.7/5 ± 0.9) were in agreement that it will impact their future practice.

Additionally, pharmacists were asked about their perspectives on the role of patients in escalating the integration of PG testing. They suggested that increasing public awareness of PG beneficial value would help to enhance their ability in implementing the services, with an average rating of 3.5 ± 0.7 on a Likert scale where 1 is very unlikely and 5 is highly likely.

### 3.5. Willingness and Confidence

**Figure 4:**
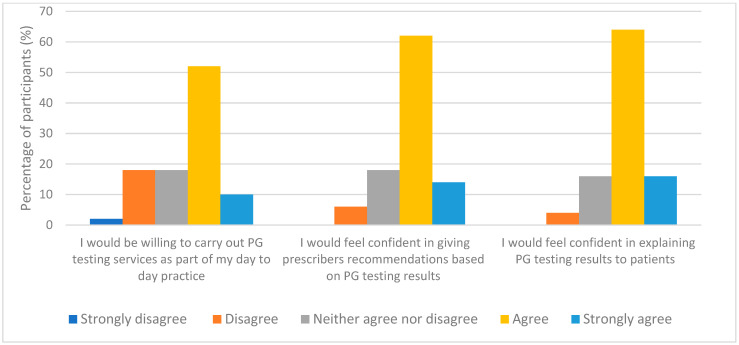
Participants responses with the assumption that they have already received adequate training (*n* = 50).

When participants were asked to rate their confidence in delivering PG testing, almost half of the respondents 52% (26/50) expressed a lack of confidence in offering this service while only 6% (3/50) said they were “fairly confident”. The Fisher Exact Test was performed to evaluate if there was an association between their current levels of confidence and their understanding for PG topic. It was found that the better their comprehension, the higher their levels of confidence; with a statistically significant *p*-value of 0.003 (significance established at *p* < 0.05). However, the picture changed when they were asked to rate their confidence and willingness to deliver PG testing assuming they had received an adequate level of PG training, with over 60% of respondents either agreed or strongly agreed that they would be willing to carry out PG testing services, as shown in Figure 4.

## 4. Discussion

Undoubtedly, community pharmacists can have an important role in successfully implementing pharmacogenomic (PG) testing service, however as the results indicate, there are currently several factors impeding the progress and implementation of this service.

It is important that PG education and training are provided at a sufficient level, so pharmacists are equipped with the necessary knowledge and skills. The study examined the current perception and extent of PG knowledge among community pharmacists in South London and revealed a low level of knowledge in PG with an average of 42% answering the knowledge questions accurately and 35% identifying drugs that have the potential for PG testing. This aligns with another study by Jarrar et al. [20], involving 370 pharmacists which showed that only 38% of respondents could correctly list medications requiring PG testing. Furthermore, a correlation was observed between the duration of participants’ registration and their current level of understanding of the PG topic. This suggests that the initial university education and training is evolving to include more PG content placing those that qualified many years ago be at a disadvantage. A similar outcome was reported in another study which showed that the age of respondents and number of years in practice were associated with lower knowledge scores [21].

Although 40% of pharmacists learnt about PG at their university studies, there appeared to be a variation in the effectiveness of this teaching. This could be due to inconsistencies amongst pharmacy schools’ curriculums within the country or even globally. The results can be compared to a study conducted in Australia, which reviewed the curriculum of 22 pharmacy degree programs. The courses’ profiles were electronically screened for key terms such as ‘genetics’ and ‘pharmacogenomics. The results showed that 82% of the programs incorporated PG into their curricula, with 18% offering PG courses as standalone and 45% contained PG related material in other science courses. However, the scope of training was limited, and schools of pharmacy were covering basic concepts, such that teaching was at an ‘understand’ level [22]. This can signify the requirement for pharmacy schools to teach PG at a consistent level that equips pharmacists with the confidence and knowledge needed to effectively deliver this service. In addition, it is crucial to bridge the gap in knowledge among current practicing pharmacists through training, as per the findings of the current study, pharmacists are willing to receive both types of training, online or face to face formats.

The acceptance of pharmacist recommendations by prescribers is an essential constituent of clinically driven pharmacy services that is often overlooked. Only a small proportion of pharmacists reported their recommendations being frequently actioned by prescribers; nevertheless, respondents were more optimistic about prescriber acceptance of recommendations regarding PG. The findings relate to a study that looked at the prescriber acceptance of pharmacists’ recommendations concerning statin therapy for primary prevention and the use of high-risk medications. The authors found that only 35% of recommendations made by pharmacists were accepted by prescribers [23]. There could be various reasons for that including mistrust between prescriber and pharmacist, poor communications, pharmacists not giving clear rational for their recommendation, or prescribers lacking awareness on the expertise and scope of pharmacists’ knowledge. On the contrary, when assessing prescriber acceptance of pharmacist recommendations based on PG test results, a different trend emerged. Ferreri et al. [24], showed that 100% of recommendations related to PG testing results were ultimately accepted by prescribers. It can be deduced that prescribers may have more of an acceptance towards recommendations specifically related to PG compared to general ones. 

Most pharmacists came to believe that taking samples would be part of their role in PG testing, however it was seen that respondents were not as keen on making recommendations on treatment choice, dosage changes, monitoring, or interpreting results. This could indicate a potential lack of knowledge or self-confidence in their capabilities as clinical pharmacists, as well as not fully understanding the impact they can have in executing this service in a community pharmacy setting. A randomised controlled trial examined the outcomes of pharmacogenomic guided antidepressant therapy compared to standard treatment in a community pharmacy setting. Recommendations that were made included, dose adjustments, medication change, new medication added to regimen and medicine adherence. The investigation showed that pharmacist-led pharmacogenomic guided therapy showed greater improvements in mental health conditions compared to those receiving standard treatment [25]. This demonstrated that pharmacists possess the capabilities and understanding to offer more than taking samples and can really make a difference to patients by improving their therapeutic outcomes.

The results clearly showed that many barriers are in place that may prevent community pharmacists from successfully implementing the PG testing service and time constraints identified as the greatest obstacle. Community pharmacies in England have experienced an increase in number of prescriptions dispensed and expansion of the services offered in particular with the introduction of the new community pharmacy contractual framework which have amplified the workload for pharmacists [26].

Cost and funding were also regarded as a significant barrier hindering the implementation of PG services in community pharmacy. This is a valid concern as the economic analysis has shown that up to 85% of community pharmacies will be in financial deficit by 2024 if the current funding arrangements continue; moreover, 52% of pharmacy owners are planning to sell their business [27]. This goes to show there is underfunding in the sector and if PG testing is to become a reality within the community pharmacy field, government funding must expand to allow its continual operation, or alternatively private funding options could be considered. Lack of motivation was also seen to be a sizable barrier, and this could further be related to the increasing workload and the risk of burnout, as demand for clinical services from pharmacy staff is set to increase by 90% within 5 years of the current pharmacy contract [28]. On the other hand, the NHS Long Term Workforce Plan, through training programs, aims to expand the role of pharmacy technicians, enabling them to contribute to the workload and accordingly freeing up pharmacists’ time to provide clinical services [29].

Although standardisation of clinical guidance was not perceived as a significant barrier to the implementation of a PG service, there is a consensus that despite scientific advancements in understanding of how genetic variations affect medicines, there is a lack of understanding on how to use the results of genetic screening to guide medication selection [30]. Having access to clinical guidance, decision support pathways and standard operation procedures will aid in translating these results, hence allowing clinicians to optimise treatment plans based on PG results.

Current levels of confidence were shown to be low amongst respondents; The results could further be compared to a study looked at pharmacists’ current views toward PG testing. It was shown that 95% of individuals would be willing to endorse PG testing to prospective patients, however only 8% currently felt confident in explaining the results of PG testing to patients [31]. This suggests that whilst optimism and drivers are present, the current limited levels of PG knowledge and confidence may hinder them from being able to utilise their full expertise and potential.

The study had identified barriers to the implementation of PG service; however, with strategies, investment and drawing from the experiences of other countries that have integrated the PG, progress can be made. For example, the Netherlands has been a pioneering in the PG field, particularly through the establishment of the PG Consortium and the PG Working Group that have published a range of guidelines focusing on gene-drug interactions providing interpretation and medicine recommendations [32].

Examining other services provided by community pharmacists, for example, the contraceptive prescribing service initially encountered objection and reluctance in acceptance in some areas in the United States, but pharmacists have proven their worthiness through their counselling skills, allowing wider access to care, and in patients welcomed having this service readily available at the pharmacy [33]. Education at the university level and training programs have been developed to successfully prepare for this service. All these factors had led to a shift in perception and acceptance for the role of pharmacists in contraceptives supply. Simialry in England, certain named contraceptives have been added to the allowable sale items subject to pharmacist consultation, and moreover “supply contraceptive” initiative as a pilot service under the discretion of an agreement “patient group directions” is under evaluation [34]. This example demonstrates that despite the challenges, with careful planning, pharmacists can widen their scope of practice and add value to the services and patient care.

PG teaching within pharmacy courses is embedded within various subjects, However, having a dedicated course explaining the science of the PG and its clinical applications can help pharmacy students to graduate with solid foundation and become more confident in that field. A study by Higgs et al. (2008) found that 84% of the medical schools in the UK that participated in their study provided only 1–2 h of PG relevant teaching [35]. This also echoes the findings of other studies where pharmacy students’ surveys indicated the need for an updated pharmacy curriculum that places a stronger emphasis on PG and integrates PG courses into the overall curricula [36,37]. Building foundation for precision medicine from the early years of education is essential, nevertheless, bridging the gap for current practicing pharmacists is useful and can be a quick solution to the move towards genetic-informed therapy.

The study had some limitations. The primary constraint was the limited number of respondents in the survey. It would have been advantageous to have a larger sample of participants to provide more representative views and further support the current observations. Factors that may have contributed to the low participation rate included the availability and workload of pharmacists. Additionally, the survey included a total of 36 questions, some of which were intricate, especially in the knowledge section. Consequently, some pharmacists possibly have found it challenging to complete the survey or felt that it could potentially expose gaps in their knowledge. Secondly, none of the participating pharmacists had prior experience in delivering the PG services, which was reflected in their responses, showing they did not have a mature vision about the service and their potential role in it. Finally, including a wider participation from different regions, such as those outside London, is valuable as those pharmacies would have been part of different integrated care systems leading to different experiences and perspectives regarding their readiness to deliver genomic testing services. Therefore, targeting a larger and diverse group of participants would allow for more generalised results.

## 5. Conclusions

The study revealed that not all community pharmacists are prepared for the implementation and delivery of a PG testing service. A large barrier is confidence and that may be associated with factors such as increased workload, poor knowledge, and time constraints. Consistent and uniform education and training is required to allow all community pharmacists to be practicing on the same level with similar skill sets and enable them to play a vital role in genomics-informed treatment.

## Figures and Tables

**Table 1:** Demographics details of study participants.

Characteristics	Number of Participants (*n* = 51) *n* (%)
**Age Group**	
23–32	25 (49.0)
33–42	12 (23.5)
43–52	10 (19.6)
53–62	4 (7.8)
63+	0 (0)
**Gender**	
Male	22 (43.1)
Female	29 (56.9)
Other/prefer not to say	0
**Number of years in practice**	
<1	1 (2.0)
1–4	12 (23.5)
5–10	16 (31.4)
11–15	8 (15.7)
16–20	3 (5.9)
20+	11 (21.6)
**Type of pharmacy**	
Independent pharmacy	17 (33.3)
Small pharmacy chain	15 (29.4)
Large pharmacy chain	17 (33.3)
Small chain and independent pharmacy	2 (3.9)
**Role within pharmacy**	
Manager	6 (11.8)
Responsible pharmacist	15 (29.4)
Second pharmacist	4 (7.8)
Locum	19 (37.3)
Relief	2 (3.9)
Mix of the roles above	5 (9.8)
Superintendent	0 (0)

**Table 2:** Percentage of pharmacists responding correctly regarding the current PG testing status of common drugs.

Drug	Status	% of Pharmacists with Correct Response (*n* = 49)
Abacavir	Current PG testing available	61% (30/49)
Carbamazepine	Suitable for future PG testing	33% (16/49)
Clopidogrel	Suitable for future PG testing or Current PG testing available	33% (16/49) 6% (3/49)
Co-amoxiclav	Neither	51% (25/49)
Codeine	Suitable for future PG testing	17% (8/49)
Fluorouracil	Current PG testing available	47% (23/49)
Sertraline	Suitable for future PG testing	47% (23/49)
Simvastatin	Suitable for future PG testing	45% (22/49)

## Data Availability

Please contact the corresponding author for questions regarding data.
